# The Effects of Zr Doping on the Optical, Electrical and Microstructural Properties of Thin ZnO Films Deposited by Atomic Layer Deposition

**DOI:** 10.3390/ma8105369

**Published:** 2015-10-27

**Authors:** Stephania Herodotou, Robert E. Treharne, Ken Durose, Gordon J. Tatlock, Richard J. Potter

**Affiliations:** 1 Centre for Materials and Structures, School of Engineering, University of Liverpool, Brownlow Hill, Liverpool L69 3GH, UK; tatlock@liverpool.ac.uk (G.J.T.); rjpott@liverpool.ac.uk (R.J.P.); 2 Stephenson Institute for Renewable Energy, University of Liverpool, Liverpool L69 7ZF, UK; treharne@liverpool.ac.uk (R.E.T.); dph0kd@liverpool.ac.uk (K.D.)

**Keywords:** ZnO, Zr doped ZnO, ALD, TCO, optical gap blue-shift

## Abstract

Transparent conducting oxides (TCOs), with high optical transparency (≥85%) and low electrical resistivity (10^−4^ Ω·cm) are used in a wide variety of commercial devices. There is growing interest in replacing conventional TCOs such as indium tin oxide with lower cost, earth abundant materials. In the current study, we dope Zr into thin ZnO films grown by atomic layer deposition (ALD) to target properties of an efficient TCO. The effects of doping (0–10 at.% Zr) were investigated for ~100 nm thick films and the effect of thickness on the properties was investigated for 50–250 nm thick films. The addition of Zr^4+^ ions acting as electron donors showed reduced resistivity (1.44 × 10^−3^ Ω·cm), increased carrier density (3.81 × 10^20^ cm^−3^), and increased optical gap (3.5 eV) with 4.8 at.% doping. The increase of film thickness to 250 nm reduced the electron carrier/photon scattering leading to a further reduction of resistivity to 7.5 × 10^−4^ Ω·cm and an average optical transparency in the visible/near infrared (IR) range up to 91%. The improved n-type properties of ZnO: Zr films are promising for TCO applications after reaching the targets for high carrier density (>10^20^ cm^−3^), low resistivity in the order of 10^−4^ Ω·cm and high optical transparency (≥85%).

## 1. Introduction

Doped zinc oxide is of interest as a transparent conductive oxide (TCO), due to its low resistivity (≤10^−3^ Ω·cm), high transparency (>80%) and wide bandgap (3.37 eV [1]). The abundance, and hence low cost of the major constituents, makes this an attractive alternative to indium tin oxide (ITO), which contains relatively scarce and expensive indium. TCOs must have low resistivity (≤10^−3^ Ω·cm [2]), high transparency (>80% in the visible range [3,4]) and high carrier concentration (≥10^20^ cm^−3^ [5]). ZnO tends to be intrinsically n-type and can be readily doped to degeneracy, hence providing high conductivity. The high carrier density resulting from the degenerate doping could also induce optical gap increases due to band filling effects (*i.e*., Burstein-Moss effect), which enhances transparency in the short wavelength region. The dopants used in ZnO should be shallow donors that provide extra ionized electrons. Dopants such as B [6,7], In [8,9], Co [10], Zr [11,12], Ge [13], Hf [14], Sn [15] have been studied, while the most common dopants are Al [16,17] and Ga [3,18,19,20]. The reported electrical and optical properties for doped ZnO are being improved by using different dopants in order to compete with ITO, which has a resistivity in the order of 10^-4^ Ω·cm and transparency ≥85% [21].

Zirconium was chosen as the dopant in the current work due to its abundance, comparable ionic size to Zn and because it can potentially act as double donor providing up to two extra free electrons per ion when substituted for Zn^2+^ [22]. The close match between the ionic size of Zr^4+^ compared to Zn^2+^ [23] (*i.e*., 0.745 Å for Zr and 0.740 Å for Zn [22]) should help to minimize lattice distortion, which is often observed with other dopants such as Al [15]. Al can sit in interstitial positions in ZnO due to its small ionic radius [24], which can have the side effect of reducing interstitial Zn defects that act as native donors. This is avoided if the dopant ions are comparable in size to Zn ions as the dopant can readily sit on Zn sites, which is the case with Zr^4+^. An additional advantage of using Zr as a dopant is that it does not readily bond with Zn atoms, hence is unlikely to form secondary intermetallic phases [25]. A number of publications report on Zr doped ZnO deposited by spray pyrolysis [12,26], low temperature co-precipitation method [27], sol-gel [15,28,29,30], direct current (DC) magnetron sputtering [31,32,33], pulsed laser deposition (PLD) [34], and ALD [11]. 

ALD was used in the current study to produce modulated (delta) doped ZnO during the deposition itself, which is considered an advantageous method for accurate control of the carrier concentration. The self-limiting nature of ALD also provides excellent control over film thickness, good uniformity and conformality, while the relatively low growth temperature permits the use of temperature sensitive substrates such as polymers. A comprehensive report is presented in this paper about the limits in electrical properties of Zr doping in ZnO thin films, and how this affects the optical properties. Lin *et al*. [11] reported a study on Zr doped ZnO films deposited by ALD, covering topics such as the conductivity improvement and bandgap increase after doping, while focusing on the effect of annealing the doped samples. The current study builds up on this study by exploring how the Zr doping incorporated in the lattice, identifies possible causes of the carrier density decrease at high doping, investigates the doping effect on the grain growth, and finally examines the reason for the optical gap increase after doping.

## 2. Experimental Section 

**Figure 1:**
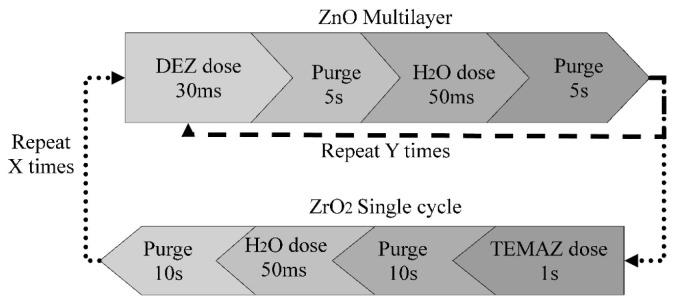
Schematic of the atomic layer deposition (ALD) process used to deposit Zr doped ZnO.

The deposition was carried out in an Oxford Instruments OpAL ALD reactor (Oxford Instruments, Bristol, UK) at 200 °C using diethylzinc (DEZ) and tetrakis-ethylmethylamino zirconium (TEMAZ) as Zn and Zr sources respectively (supplied by SAFC Hitech), with water vapour as a co-reactant. Each precursor was delivered via vapour draw, with DEZ and H_2_O sources held at room temperature, while the TEMAZ was heated at 95 °C. Films were deposited on sodalime glass microscope slides (cleaned with isopropanol and dried with nitrogen), and on virgin test grade Si (100) wafers. The growth rates of ZnO and ZrO_2_ individually at 200 °C were 1.87 and 0.65 Å/cycle respectively. Zr doped ZnO with target doping ratios between 0 and ~10 at.% were deposited using an ALD delta doping methodology similar to the one reported by Chalker *et al.* [13]. As illustrated in Figure 1, ZnO multilayers were deposited by repeated ALD cycles, interspersed by one ALD cycle of ZrO_2_. The doping percentage was calculated from the number of doping cycles and the number of Zr atoms per ZrO_2_ monolayer. The film compositions presented in this study are nominal. The overall thickness of the layer was then controlled by repeating this “master” cycle multiple times. To improve the quality of the films, an ultra-thin (~2 nm) buffer layer of Al_2_O_3_ was deposited on the substrates by ALD (using 20 cycles of trimethyl-aluminum and water) prior to the deposition of the doped ZnO. The Al_2_O_3_ process promotes uniform deposition of the target films (probably due to self-cleaning), and is also believed to enhance the optical performance, since it provides transverse optical confinement with minimum absorption loss due to its wide energy gap [35]. 

Films grown on glass were used for all subsequent optical analysis, while films on Si (100) were used for microstructural analysis. Film thickness was assessed using a Rudolph Research Auto-EL-IV Ellipsometer (Rudolph Research Analytical, Hackettstown, NJ, USA) operating at 633 nm. The microstructure was analysed by atomic force microscopy (AFM) (Bruker Corporation, Billerica, MA, USA) using a Bruker Multimode 8. The microstructure was calculated from the X-ray diffraction (XRD) patterns obtained using a Rigaku Miniflex Diffractometer (Rigaku Corporation, Tokyo, Japan) in the Bragg-Brentano geometry and using a Cu Kα X-ray source. The chemical state of the films was determined by X-ray photoelectron spectroscopy (XPS) using a FISONS VG Escalab MKII Scientific XPS (Fisons plc, Ipswich, UK), with Al Kα radiation. The electrical properties of the films were assessed by four-point-probe (4PP) and by Hall effect measurements. The 4PP measurements were carried out using a homebuilt system comprising of a Lucas Signatone Corp SP4 Probe head (Lucas Signatone Corp., Gilroy, CA, USA) and a Keithley 2400 series sourcemeter (Keithley Instruments Inc., Solon, OH, USA), while the Hall measurements used the same sourcemeter attached to a BioRad Hall probe station with a 0.3 T fixed magnet. Hall measurements were carried out using van-der-Pauw sample configuration with indium contacts. The optical properties of the films were assessed by UV-Vis transmission using a Shimadzu SolidSpec-3700DUV dual beam UV-Vis spectrophotometer (Shimadzu Corp., Kyoto, Japan) and by photoluminescence (PL) using a Horiba JY LabRam HR HR800 confocal Raman microscope (Horiba Ltd., Kyoto, Japan) fitted with a He-Cd UV laser (325 nm).

## 3. Results and Discussion

### 3.1. Effect of Doping and Film Thickness on the Microstructure and Electrical Properties.

**Figure 2:**
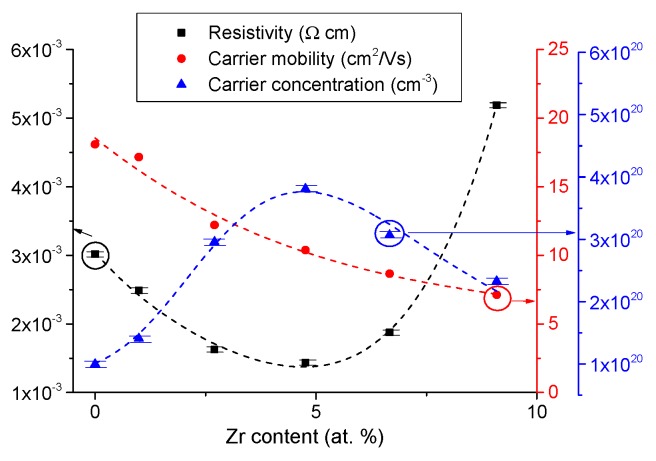
Zr doping dependence of resistivity, carrier concentration and mobility for ZnO films ~85 nm thick.

Figure 2 shows the effect of doping on the resistivity, carrier density and mobility as a function of Zr doping for films that are ~85 nm thick. The carrier density increases from 1.0 × 10^20^ to 3.81 × 10^20^ cm^−3^ as the doping is increased from zero to 4.8 at.%, leading to a decrease in resistivity from 3.02 × 10^−3^ to 1.44 × 10^−3^ Ω·cm. 

**Figure 3:**
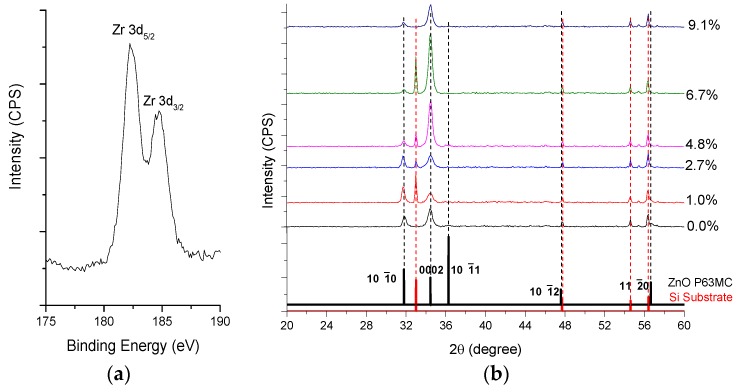
(**a**) X-ray photoelectron spectroscopy (XPS) spectrum for the Zr 3d peaks for 250 nm thick film with 4.8 at.% Zr doping; (**b**) X-ray diffraction (XRD) patterns of Zr doped ZnO films.

The increase in carrier concentration is mostly attributed to the ionisation of the Zr atoms on Zn-sites, where the Zr^4+^ ions replace Zn^2+^ ions, thus donating two extra electrons to the system [28]. This interpretation is supported by previously reported theoretical studies that suggest that Zr dopant prefers Zn-site substitution (Zr_Zn_) in the bulk of the film rather than locating at the surface as is the case of Cu and Ag [36]. First principles calculations have also shown that Zr_Zn_ has lower formation energy than interstitial (Zr_i_) and oxygen vacancy (Zr_O_) positions [23]. In the current study, XPS reveals evidence that Zr is substitutional to Zn sites (Zr_Zn_), which indicates that Zr is in its ionic state (Zr^4+^) in the Zr-O structure. The binding energies of Zr3d_5/2_ and Zr3d_3/2_ are 182.4 and 184.7 eV respectively (Figure 3a) [37]. In addition, XRD provides evidence that Zr is not forming interstitial defects as an increase in the crystal alignment is observed at high doping levels (Figure 3b), suggesting that the hexagonal structure is maintained after doping. Interstitial Zr is expected to promote amorphization [12]. The XRD also indicates that very little lattice distortion is induced by Zr doping. A nominal lattice constant increase of ~0.01 Å with 4.8 at.% doping (*i.e*., ${\text{d}}_{\left(10\bar{1}0\right)}$=2.806 Å for 0 at.% and ${\text{d}}_{\left(10\bar{1}0\right)}$=2.814 Å for 4.8 at.%, ${\text{d}}_{\left(0002\right)}$=2.600 Å for both) suggests that Zr is not sitting on oxygen vacancy sites within the lattice as this would have created larger lattice distortion due to the large repulsive forces between Zr and Zn atoms [23]. 

The ionic bonding between Zn^2+^ and O^2−^ suggests that the free electrons causing the n-type semiconductor behavior in the doped ZnO originate from the intrinsic donors (zinc interstitials and oxygen vacancies), hydrogen acting as donor [38] and the donor dopants (Zr^4+^). The hydrogen concentration is not expected to change significantly as Zr is added and hence is not expected to contribute significantly to the increase in carrier density due to doping. The main increase in carrier density with doping is attributed to Zr^4+^ acting as an electron donor, potentially providing up to two extra carriers per ion. If each Zr^4+^ dopant ion were acting as a double donor, then the addition of 4.8 at.% Zr to the ZnO would theoretically lead to an increase of ~4.1 × 10^21^ cm^−3^ in the carrier density. In reality, Hall effect measurements indicate that the addition of 4.8 at.% Zr causes the carrier density to rise by only ~2.8 × 10^20^ cm^−3^, which possibly suggests the formation of neutral defects during doping and segregation of Zr at grain boundaries.

**Figure 4:**
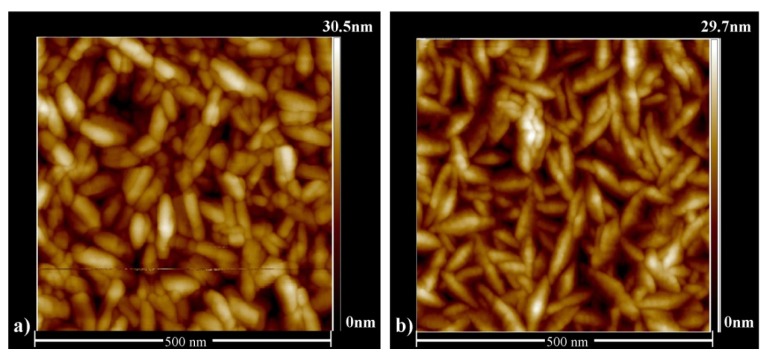
Atomic force microscopy (AFM) of (**a**) Un-doped; (**b**) 4.8 at.% doped ZnO films with a nominal thickness of ~250 nm.

As the doping level increases beyond 4.8 at.%, the carrier concentration begins to decrease again, resulting in an increase of resistivity. Previous studies [28,37] have attributed this high level doping behavior to the formation of segregated ZrO_2_, which inhibits effective doping of the ZnO and hence leads to a reduction in carrier concentration. The ZrO_2_ tends to cluster at grain boundaries, and this has been shown to result in suppression of grain growth and hence smaller grains [15,37]. AFM studies have been carried out on samples spanning the doping range investigated and provide evidence that grain size decreases as the doping level increases. Figure 4 shows examples of AFM for undoped and 4.8 at.% doped films (~250 nm thick) and clearly shows that the films are made-up of needle-like grains. Although it is not possible to analyze grain length using AFM due to the unknown orientation of individual grains, it is possible to study grain widths. Numerical analysis reveals that average grain width reduces from 32 nm for the undoped sample, to 25 nm for the 4.8 at.% sample suggesting that doping is indeed suppressing the grain size. This trend of grain width reduction continues as the doping level is increased. The same effect is observed for the thinner (~85 nm) films, where the average grain width reduces from 25 nm for the undoped film to 20 nm for the 4.8 at.% film and 18 nm for the 9.1 at.% film (data not shown). 

**Figure 5:**
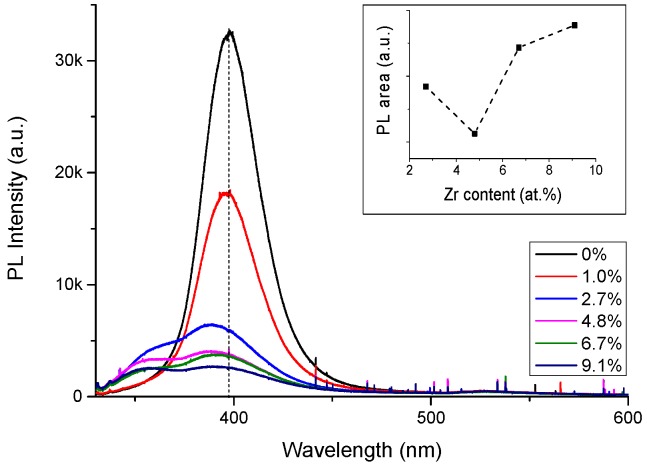
Zr doping dependence of photoluminescence (PL) emission for films ~85 nm thick grown on glass substrates. The inset graph shows the integrated area of the conduction band minimum (CBM) to the V_Zn_^0^ emission feature as a function of doping level (the feature is the shoulder on the high energy side of the main peak found by nonlinear curve fitting).

In addition to dopant segregation having a direct effect on the effectiveness of the doping, the suppressed grain size due to the doping is believed to have a secondary effect on the carrier density by reducing zinc vacancies (V_Zn_). The PL data (Figure 5) showed increased V_Zn_ concentration for the heavier doped films. The PL shows two main emission features for the doped films attributed to the recombination of carries from the conduction band minimum (CBM) to the V_Zn_^0^ state (3.14–3.15 eV) [39] and the increased band-to-band recombination energy due to Burstein-Moss effect (~3.5 eV). The CBM to V_Zn_^0^ emission feature is the shoulder on the high energy side of the main peak and is related to the zinc vacancy concentration in the films. The integrated area of this PL feature was found by a nonlinear curve fitting (Loranzian) using two peaks for all films and the data are shown in the inset graph. The intensity of the high energy peak was zero for the two lower doped films (0–1 at.%) and thus is not shown in the graph. The integrated area of the CBM to V_Zn_^0^ emission shows a slight reduction as the doping level increases from 3 to 5 at.% followed by an increase as the doping level increases up to 9.1 at.%. Zinc vacancies act as intrinsic acceptors and are easily formed in n-type materials by means of their formation energy decrease as the Fermi level increases [40]. Grain boundaries act as efficient sinks for zinc vacancies [41], hence, the concentration of V_Zn_ is proportional to the grain boundary areas. The grain boundary areas increase at high doping level would be expected to correlate to an increase of the density of zinc vacancies per unit volume. The combination of V_Zn_ and Zr^4+^ segregated at the boundaries is likely to lead to the formation of neutral defects and hence to the reduction of free electrons. The increase of the defect concentration observed in the PL data is consistent with the carrier density reduction for the heavier doped films.

**Figure 6:**
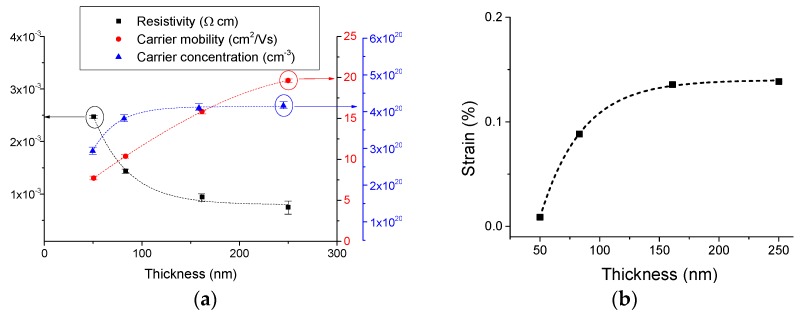
(**a**) Thickness dependence of resistivity, carrier concentration and mobility for ZnO films doped with 4.8 at.% Zr; (**b**) Thickness dependence of strain in $\left(10\bar{1}0\right)$plane for ZnO films doped with 4.8 at.% Zr.

The carrier mobility (Figure 2) decreases as the doping level increases, and this could be due to ionised impurity and possibly grain boundary scattering caused by the grain size reduction. The effect of doping concentration on resistivity (initial decrease followed by an increase), is widely reported for other doped ZnO systems, such as ZnO:Al [16], ZnO:Ge [13], ZnO:Ga [20] and ZnO:Ni [42]. Having established the Zr doping level that provides the lowest resistivity, the doping level was fixed at 4.8 at.% and the effects of film thickness were investigated. Figure 6a shows the effect of film thickness on the resistivity, carrier concentration and carrier mobility for the doped films. The mobility increases with film thickness up to a maximum value of 19.7 cm^2^/Vs It may be postulated that this behavior is consistent with an interface carrier scattering mechanism, with a higher value observed (for the thicker film) being consistent with that of the bulk material. The carrier density reaches 4.6 × 10^20^ cm^−3^, and it is largely independent of film thickness (for films >80 nm thick) as expected. The drop-off effect for very thin films (<100 nm) is attributed to the strain sensitivity of very thin films before reaching the bulk thickness.

The tensile strain measured from ($10\bar{1}0$) XRD peak positions (Figure 6b) appears to have the same increasing trend as the carrier density. Strain effects induced by the thickness changes (larger grains) could contribute to shifts in the distribution of the density of states and cause a small reduction in the bandgap [43]. In the current films the gap is expected to be reduced as the tensile strain increases with thickness, causing an increase of the carrier density. 

The combination of increasing mobility and carrier density results in an asymptotic decrease in resistivity by a factor of 3 as thickness increases from 50 to 250 nm (Figure 6a). This is consistent with previous studies of thickness effect using other dopants such as Ga [18] and Cu [44].

The lowest resistivity value achieved in this work was 7.5 × 10^−4^ Ω·cm for a 250 nm film, with resistivity remaining below 10^−3^ Ω·cm for films >150 nm. This is in the desired range for TCO applications. This value is lower than other published values for Zr doped ZnO films fabricated by magnetron sputtering for which the resistivity of films 200–300 nm thick was ~2 × 10^−3^ Ω·cm [31,37], and it is also below the value of vacuum annealed 450 nm thick film which achieved 9.8 × 10^−4^ Ω·cm [33]. It is also comparable to the lowest resistivity values published for other doped ZnO coatings grown by ALD such as Al doped (7.7 × 10^−4^ Ω·cm) [17] and Ga doped films (8 × 10^−4^ Ω·cm) [20].

### 3.2. Effect of Doping on the Optical Properties.

**Figure 7:**
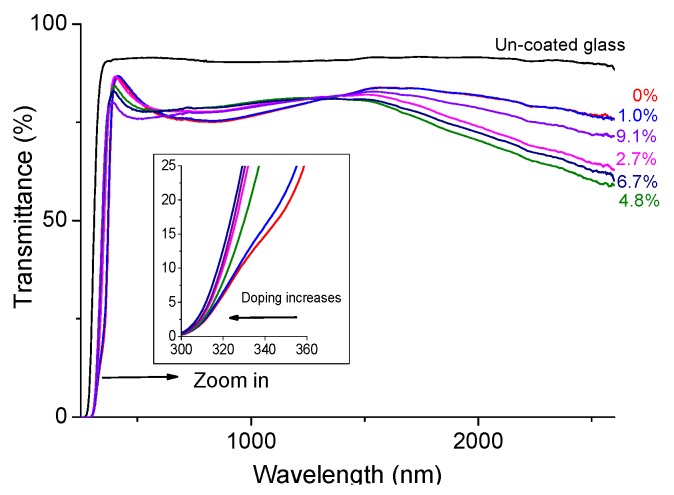
Transmission spectra of ~100 nm thick Zr doped ZnO films as a function of the atomic concentration of Zr.

Figure 7 shows the raw optical transmission spectra (*i.e*., before normalisation) of ~85 nm doped ZnO films with various fractions of Zr. Films with 1 to 4.8 at.% Zr have a high normalised transparency, averaging 87%–88% in the visible (380–780 nm) and 89% in the near IR (780–1400 nm) range (normalisation was done by scaling the transmission values by the coefficient necessary to increase the experimental value for uncoated glass to 100%). The normalized transparency in the visible spectrum was at its maximum (91%) for a thickness of 161 nm, and in the near IR region reached 91% with a thinner film (50 nm). The high transparency and low resistivity results in high values of the figure of merit calculated by the formula derived by Haacke [45] as expressed in Equation (1).

(1) $$F_{TC}=\frac{T^{10}}{R_{s}}$$

where *T* is the average transmittance in the visible region and *R_s_* is the sheet resistance. The value for the thicker doped film (250 nm) was 0.93 × 10^−2^ Ω^−1^ while for a thinner one (83 nm) was at 0.14 × 10^−2^ Ω^−1^. For comparison ITO typically has 1.19 × 10^−2^ Ω^−1^ [46]. The results of the current study are also promising compared to other doped ZnO films deposited by ALD, such as 100 nm Al-doped ZnO (*F_TC_* = 0.10 × 10^−2^ Ω^−1^) [47], 180 nm In-doped ZnO (*F_TC_* = 0.12 × 10^−2^ Ω^−1^) [48], 100 nm Ti-doped ZnO (*F_TC_* = 0.12 × 10^−2^ Ω^−1^) [49] and 200 nm Hf-doped ZnO (*F_TC_* = 0.36 × 10^−2^ Ω^−1^) [14].

**Figure 8:**
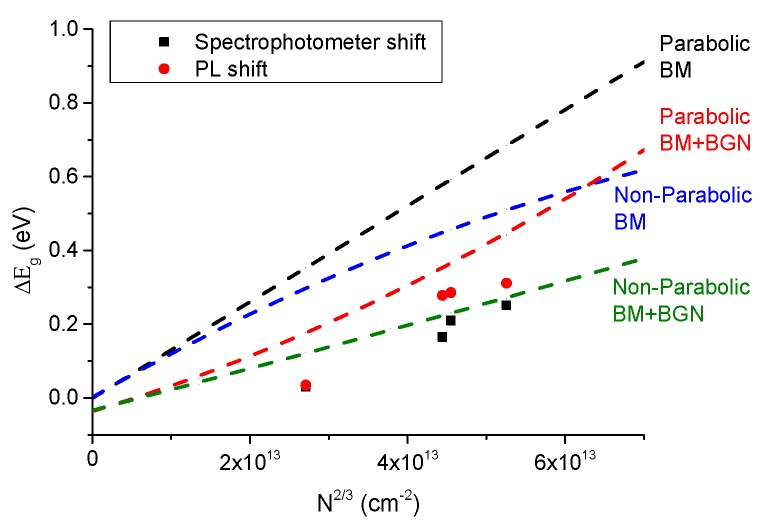
Optical gap difference between the doped and un-doped films as a function of carrier concentration.

As expected, the long wavelength plasma edge of the transmission spectra is affected by doping as the plasma frequency is proportional to the square root of the carrier concentration. The optical gap was measured experimentally by both optical transmission and by PL. In transmission the values for undoped and 4.8 at.% doped samples were 3.27 and 3.53 eV (*ΔE *= 0.26 eV) which are in close agreement with PL values of 3.21 and 3.52 eV (*ΔE *= 0.31 eV). The slight differences are comparable with the expected difference between absorption and emission processes. Figure 8 shows a direct relationship between the blue shift in the optical gap and carrier concentration (controlled by doping). The PL shift represents the band-to-band recombination energy shift and the spectrophotometer shift represents the gap estimated using Tauc plots.

The blue shift of the optical gap for low doping levels can be explained by a combination of Burstein-Moss (BM) [50] effect due to degenerate band filling of the conduction band (*i.e*., when *n *> 3.7 × 10^19^ cm^−3^ [51]) and bandgap renormalisation (*i.e*., narrowing) due to many body effects from the increased carrier concentration. To confirm those effects, mathematical models were applied to calculate the effective masses of parabolic (*i.e*., m* = 0.28 *m_0_* [4]) and non-parabolic conductions bands [52] and to estimate the shifts due to BM [53] and bandgap narrowing (BGN) [54] as expressed in Equations (2) and (3) respectively (Figure 8).

(2) $$∆E_{BM}=\frac{{\text{ħ}}^{2}}{2m^{*}}{\left(3{\text{π}}^{2}n\right)}^{2/3\;}$$

(3) $$∆E_{BGN}=\frac{e^{2}}{2{\text{πε}}_{0}{\text{ε}}_{r}}{\left(\frac{3}{\text{π}}n\right)}^{1/3\;}$$

The experimental results agree reasonably well with the non-parabolic band model with both effects. The non-parabolic CB was due to the band deformation by the high number of free electrons. Hence, by modulated Zr doping the optical gap can be tuned in a predictable way.

## 4. Conclusions 

ALD grown ZnO: Zr films showed that doping offers control over resistivity reduction up to 4.8 at.% due to the extra ions offered by the substitution of Zr^4+^ to Zn^2+^. At heavier doping the carrier concentration was reduced due to Zr segregation to grain boundaries consistent with the suppression of grain growth, and the formation of neutral defects at the boundaries (increase of zinc vacancies). The resistivity was decreased as thickness increasing (7.5 × 10^−4^ Ω·cm) leading to scattering reduction. The remaining decrease was accompanied by a carrier density increase (4.6 × 10^20^ cm^−3^) influenced by the tensile strain. The optical gap of the degenerate films could be enlarged up to 3.5 eV due to the net effects of the Burstein-Moss shift and bandgap renormalisation. The transparency of the doped films was up to 91% in the visible and near IR regions. The properties resulted in a high figure of merit (0.93 × 10^−2^ Ω^−1^) that is close to figures of merit for ITO films (1.19 × 10^−2^ Ω^−1^ [46]) which is the material targeted to be replaced. This figure is higher than other doped ZnO films deposited by ALD, such as In-doped ZnO (0.12 × 10^−2^ Ω^−1^) [48] and Hf-doped ZnO (0.36 × 10^−2^ Ω^−1^) [14]. As a result, the Zr-doped ZnO films grown by ALD on sodalime glass can find applications as TCOs with properties that comply with the targets for high carrier density (>10^20^ cm^−3^), low resistivity in the order of 10^−4^ Ω·cm and high optical transparency (≥85%).
